# E.M.D.R therapy and the theory of structural dissociation of the personality in severe interpersonal trauma of young adolescents

**DOI:** 10.1080/20008198.2017.1351207

**Published:** 2017-09-29

**Authors:** Penny Papanikolopoulos, Tessa-Ava Prattos-Spongalides

**Affiliations:** ^a^ EMDR-Europe, TACT HELLAS, Psychological Services, Athens, Greece

**Keywords:** Eye movement desensitization and reprocessing, theory of structural dissociation of the personality, interpersonal trauma, adolescents

## Abstract

In this presentation we illustrate the effects of combining eye movement desensitization and reprocessing (E.M.D.R) therapy and theory of structural dissociation of the personality (T.S.D.P) on dissociative and post-traumatic stress disorder (P.T.S.D) symptoms. We first briefly describe both theories and conclude why combining them in the treatment of severely traumatized adolescents with PTSD may be beneficial.

E.M.D.R therapy is an empirically valid treatment for P.T.S.D, based on numerous randomized controlled trials and several meta-analyses (e.g. Chen, Zhang, Hu, & Liang, 2015; Nijdam & Olff, 2016). The E.M.D.R Therapy Standard Protocol has eight specific phases.

Phase 1: History taking and building the therapeutic alliance and creating a case conceptualization based on the past, the present and future.

Phase 2: Client stabilization and preparation.

Phase 3: Assessment activation of traumatic memory network.

Phase 4: Desensitization of traumatic memory with the use of bi-lateral stimulation up to adaptive resolution while monitoring level of disturbance.

Phase 5: Installation allows an increase of connections and generalizations to positive cognitive networks.

Phase 6: Body scanning is used to monitor and clear any residual disturbing feelings in the body.

Phase 7: Closure ensures client stability at the end of an EMDR session and between incomplete sessions.

Phase 8: Reevaluation takes place at the beginning of the next session and it assesses treatment effects.

According to T.S.D.P each human being has an integrative capacity to deal with traumatic experiences. The integrative capacity entails two major mental actions, namely synthesis and realization. Synthesis can be thought of as the way one perceives, compares, differentiates and/or categorizes internal and external experiences in the present and over time. Realization is a higher level mental action that entails awareness of reality, accepting it and adapting to it. It entails (1) personification or a sense of ownership and knowing ‘this is what happened to me’ and knowing feelings and thoughts about it and (2) presentification being grounded in the present while able to integrate the past and the possibilities of the future. Knowing that this has happened in the past and the present and future is no longer dictated by the traumatic past (van der Hart, Nijenhuis & Steele, 2006). Integration can be thought of as staying in the present while describing a past whole life narrative and owning experience. Thus, the person can express and feel his/her painful experience and memories without avoiding them and allowing phobias to keep memories at bay. According to T.S.D.P, the failure to integrate traumatic experiences basically yields a structural dissociation of the personality into two or more mental systems (van der Hart et al., 2006).The three-phase oriented treatment of T.S.D.P includes: (i) history, assessment, stabilization, symptom reduction and skills building; (ii) treatment of traumatic memories; and (iii) personality reintegration and rehabilitation.

Young severely traumatized adolescents with PTSD who have been early victims of emotional, physical and sexual abuse within an interpersonal relationship and have exhibited dissociative symptoms, have been treated in the University Child and Adolescent Psychiatry Department, Athens, Greece by applying E.M.D.R therapy as a treatment intervention and the theory of T.S.D.P as a theoretical and conceptual tool for understanding the presenting dissociative symptoms. The theory of T.S.D.P was utilized to conceptualize the cases in terms of dissociative and P.T.S.D symptoms as they were measured at baseline, during therapy and at the end of therapy to assess change. We observed that their P.T.S.D and dissociative symptoms would decrease when they felt safe and were able to trust their own bodily reactions and emotions. Following a prolonged stabilization phase the adolescents were more integrated and able to reprocess their traumatic memories and put together their own life story.

In conclusion, by applying E.M.D.R therapy while being informed by T.S.D.P we may better understand and support severely traumatized adolescents who have been victims of early interpersonal abuse. While working with them can be very challenging, we saw promising results while applying E.M.D.R therapy guided by the concepts of T.S.D.P. There is promise in combining E.M.D.R therapy and T.S.D.P and future research should focus on its effects in terms of understanding dissociation of the personality following interpersonal trauma.
